# Enhancing foster care relationships through attachment-based intervention: the safe families study protocol, a randomized controlled trial of the circle of security parenting program^®^

**DOI:** 10.1186/s40359-025-02424-6

**Published:** 2025-02-08

**Authors:** Mathieu Pereira, Lauriane Sedes, Emilie Gadéa, Rebecca Shankland

**Affiliations:** 1 Laboratoire Développement Individu Processus Handicap et Education (DIPHE), Université Lumière Lyon 2, Lyon, 69000 France; 2 Centre hospitalier Emile Roux, 12 Bd du Dr Chantemesse, Le Puy-en-Velay, 43000 France

**Keywords:** Foster care, Attachment, Circle of Security parenting intervention, Resilience, Parental reflective functioning, Child maltreatment, Intervention evaluation

## Abstract

**Background:**

Children in out-of-home care constitute a vulnerable population often experiencing mental health challenges related to early adversity and placement disruptions. The Circle of Security Parenting program^®^ (COS-P) is an attachment-based intervention designed to enhance carer sensitivity and reflective competence, ultimately improving the quality of carer-child relationships.

**Methods:**

This study protocol follows a mixed-methods randomized controlled trial evaluating the effectiveness of the COS-P program for foster carers in France. A total of 70 foster carers will be randomly assigned to either the intervention group (receiving COS-P in addition to Treatment as Usual) or the waitlist control group (receiving Treatment as Usual only). Quantitative measures, including the Caregiving Composite Questionnaire, Parenting Stress Index, and the Marschak Interaction Method, will be administered at baseline and at follow-up). Qualitative data will be collected through focus groups with foster carers and COS-P facilitators, and through self-confrontation interviews with a subset of foster carers.

**Discussion:**

This study is the first to evaluate the COS-P program for foster carers in France. Findings will provide valuable insights into the program’s effectiveness in improving carer-child relationships and foster carer well-being, ultimately contributing to better outcomes for children in out-of-home care. The study will also explore potential moderators of treatment outcome, and shed light on the subjective experiences of participants.

**Trial registration:**

This trial is registered on ClinicalTrials.gov : NCT06701877.

## Background

### Fostering Well-being: addressing the needs of children in out-of-home care

In France, the child protection system currently supports 344,682 minors and young adults, with 51% placed in out-of-home care [1].

The SAFE FAMILIES study, situated within a broader research initiative focused on promoting the mental health and resilience of children placed under the care of Child Protection Services (CPS) is a collaborative effort led by Université Lumière Lyon 2 in partnership with the Département and the Clinical Research Unit of Emile Roux Hospital of Le Puy-en-Velay.

### The vulnerability of children in foster care

A robust body of international research underscores the heightened vulnerability of children in out-of-home care. These children experience elevated rates of emotional and behavioral difficulties with an estimated 50% struggling with emotion regulation.

Further, the prevalence of mental health disorders is significantly higher in this population, with 20% experiencing at least one diagnosable psychiatric disorder [2–6].

Bronsard et al. [7] revealed high comorbidity rates and an elevated risk of post-traumatic stress disorder in children in out-of-home care compared to their peers in the general population. In a study focusing on the mental health of adolescents in residential care, Bronsard et al. [8] observed that these young people often present with atypical and less direct expressions of their psychological distress, tending to minimize or normalize their suffering. Furthermore, within the French context, the public authorities reported a high prevalence of disabilities among children in care, noting a sevenfold increase compared to the general population. Of particular concern is the predominance of psychological and mental disabilities associated with behavioral disorders [9, 10].

### Exploring the pathways to vulnerability

Understanding the complex pathways that contribute to the vulnerability of children in foster care is crucial. Most of these children have endured significant adversity, including maltreatment and disruptions in their caregiving environments [11]. Such experiences expose them to Toxic Stress, characterized by frequent, strong, and prolonged activation of the body’s stress response system [12], which can have detrimental effects on neurocognitive and emotional development (e.g., impulsivity, anxiety, difficulties with attention and concentration), social participation (e.g., challenges with peer relationships, academic performance), and both physical and mental health [13].

**Fig. 1:**
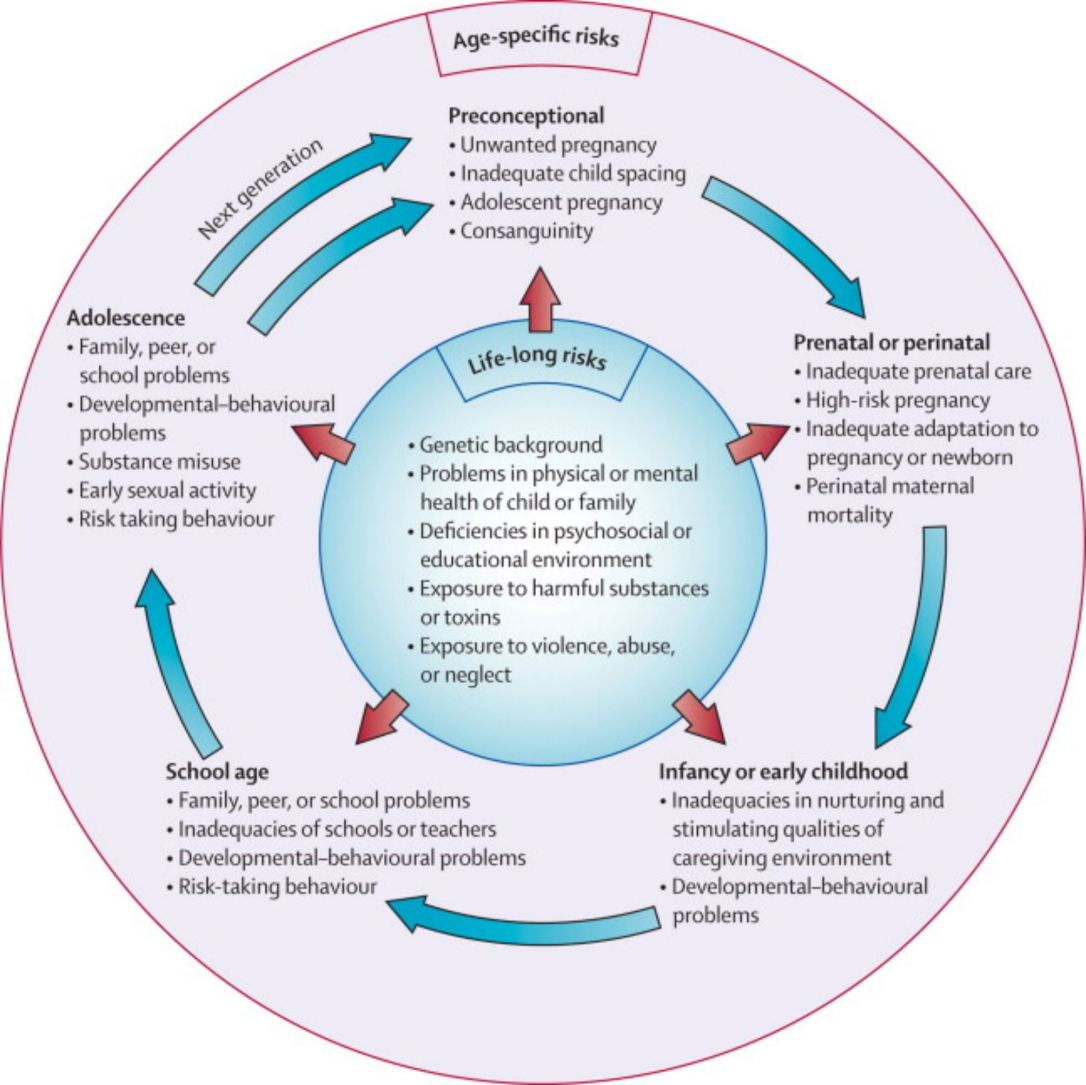
Lifecycle approach to risk factors (Kieling et al., 2011)

The psychopathological model developed by Kieling et al. [14] (see Fig. 1) provides a useful framework to understand the vulnerability of these children. This model emphasizes the accumulation of risk factors, including those related to specific developmental stages (e.g., maternal stress during the perinatal period, adolescent risk-taking behaviors), and those that are more enduring (e.g., exposure to violence, neglect, and harmful substance use).

Furthercompounding this vulnerability, the experience of being in care can itself pose challenges. Placement instability, disruptions in caregiving relationships, institutionalization, and separation from siblings all present significant risks to children’s mental health [11]. French psychiatrist David [15], a pioneer in foster care, described the potential iatrogenic effects of separation from family, even when that family context has been maltreating, as “oister care sydrome”. This syndrome can manifest through identity disturbance, attachment difficulties, loyalty conflicts, and relational and emotional breakdowns.

Finally, access to appropriate mental health services for children in foster care within the French system can be hindered by complex administrative processes and the involvement of numerous stakeholders in each child’s case [9].

### Attachment: a foundation for resilience

Attachment theory [16] provides a critical lens to understand the developmental needs of children in foster care. Attachment is a fundamental, biologically-rooted behavioral system that promotes interpersonal regulation of stress, operating from the cradle to the grave. Secure attachment is fostered when children can confidently express their emotions, particularly negative emotions like sadness or fear, to one or more consistent and attuned caregivers. This secure base allows children to explore their world with curiosity and engage in learning.

The interplay between attachment needs and the drive for exploration is central to human development. Decades of research highlight the protective power of secure attachment for mental health. A meta-analysis by Groh et al. [17] found strong associations between secure attachment and positive outcomes such as the development of social skills (d = 0.39), a reduction in externalizing problems (d = 0.31), and a lower risk of internalizing disorders (d = 0.15).

Interventions that promote sensitive caregiving and foster secure attachments are, therefore, of great clinical importance. Such interventions can be seen as a form of “psychological vaccination», particularly crucial for children in care who, due to their experiences of adversity and relational disruptions [18, 19], are more likely to have developed insecure or disorganized attachment patterns. It is essential to remember that these insecure patterns can persist even when children are placed with new caregivers who are more sensitive to their needs [20].

### Introducing the circle of security parenting® program

Developed in the United States [21], the Circle of Security Parenting^®^ (COS-P) program is a promising attachment-based intervention designed to enhance caregiver sensitivity, parental reflective functioning, and the quality of the child’s attachment to their caregiver. This manualized intervention, with both educational and therapeutic components, empowers caregivers to better understand and respond to their child’s needs, fostering secure attachment [22].

**Fig. 2:**
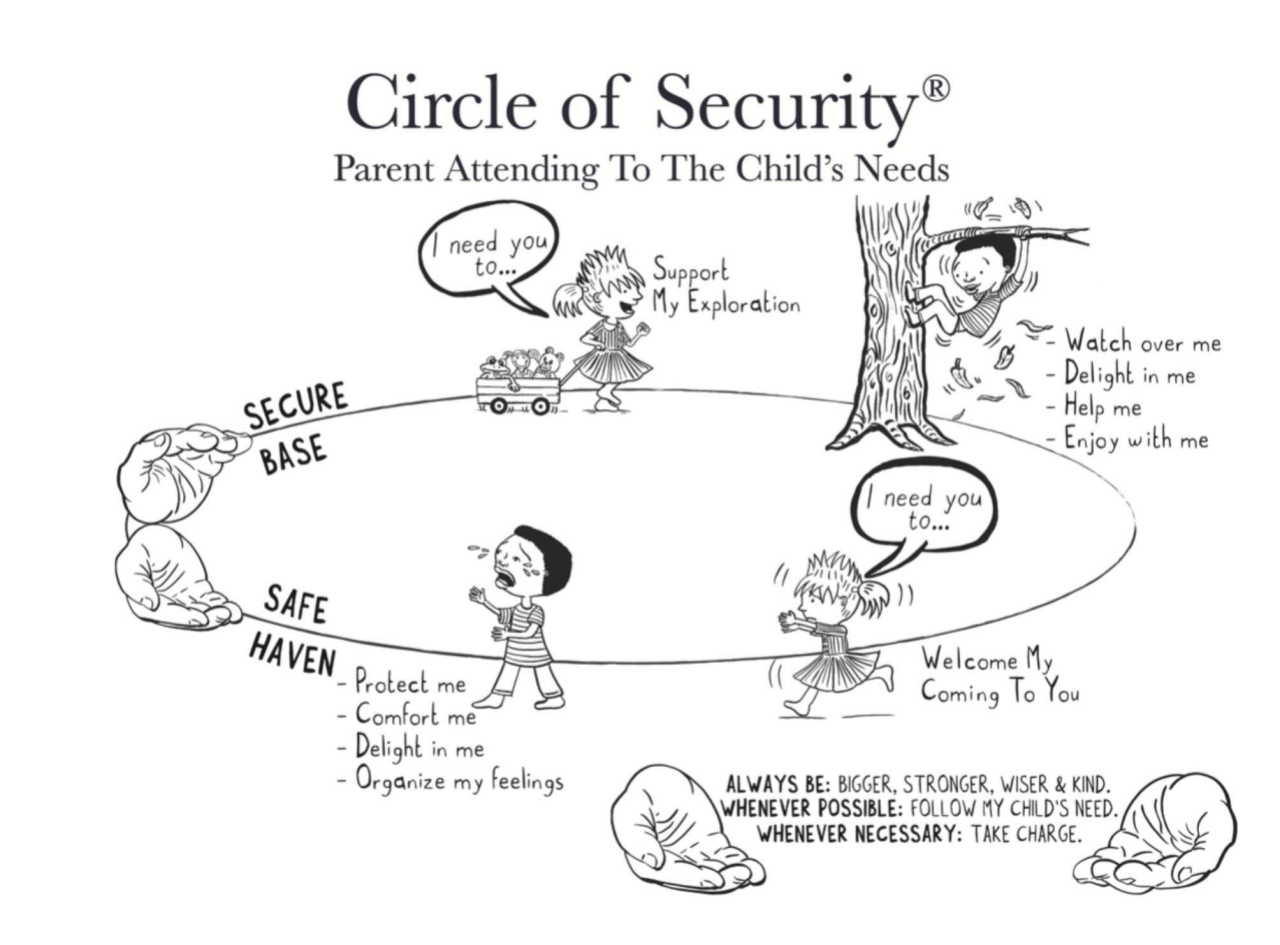
The circle of security graphic

The Circle of Security model (see Fig. 2) is central to COS-P, providing a visual representation of the child’s attachment needs and the caregiver’s role in meeting those needs.

This model highlights two essential caregiving functions:

#### Secure base

providing a safe and supportive foundation for the child’s exploration, and.

#### Safe haven

offering comfort and a secure haven when the child seeks proximity in times of stress or distress.

While the original 20-week version of the program, COS-I, includes individualized therapeutic video feedback, COS-P is a shorter, more accessible adaptation designed primarily for prevention (Cooper et al., 2009). Delivered over eight weekly group sessions (see Table 1), COS-P utilizes a manual and incorporates videos that illustrate core attachment principles and parent-child interactions. Importantly, COS-P includes an experiential dimension, encouraging participants to engage in practical exercises and reflect on their own emotional experiences within the caregiving relationship. This process of mentalization supports carers in understanding the underlying thoughts, feelings, and intentions that drive their child’s behavior.

**Table 1:** Summary of COS-P content (from: Cassidy et al., 2017; Cooper et al., 2009/2018; Woodhouse et al., 2018 ; Maxwell et al., 2021)

COS-*P* Chapter	Key COS-*P* term/s	Core content
Chapter 1 Chapter 2	COS graphic Circle map	• Basic concepts of attachment • Children’s attachment and exploration needs • COS graphic as a map for understanding parent-child interactions
Chapter 3 Chapter 4	Being with	• Importance of being with children in their emotional states • Parent coregulating the child while the child develops their own capacity for emotion regulation
Chapter 5	Shark music	• How defensive processes (often based in the parent’s childhood experiences) can influence parents’ experience of and response to their child’s needs • Importance of parents reflecting on parenting struggles to enable more supportive responding
Chapter 6	Mean, Weak, Gone	• Hostile, helpless and neglectful parenting • Disorganized attachment • Importance of reflection to help keep caregiving in balance
Chapter 7	Rupture & Repair Time in	• Importance of relationship repair following inevitable ruptures in caregiving • How repair supports the long-term development of child emotion regulation and positive relationship capacities • “Time in” as a strategy for parent–child relationship repair
Chapter 8		• Summary of core content • Participant reflection on key learnings • Celebration of program completion

Research on COS-I has demonstrated its efficacy in improving caregiver capacities and child attachment security [23], and emerging evidence suggests the potential of COS-P as well:

Cassidy et al. [24], in a study of 141 mother-child dyads in the US, found that COS-P led to a significant reduction in insensitive maternal behaviors. Also Maxwell et al. [25] observed a significant improvement in parental reflective functioning among 221 Australian mothers of children aged from 0 to 6 years of age who participated in COS-P. Building on these promising findings, recent research has begun to explore the application of COS-P within the child protection system: Bisaillon et al. [26] found that COS-P increased reflective functioning in residential care workers. Krishnamoorthy et al. [27] reported positive outcomes for Australian foster families who participated in COS-P, including reduced carer stress, improved family relationships, and a decrease in the foster children’s emotional and behavioral difficulties.

## Methods and design

### Study aims and hypotheses

The SAFE FAMILIES study is the first evaluation in France of the Circle of Security Parenting program^®^ (COS-P) for foster carers. This attachment-based intervention is designed to enhance caregiver sensitivity and parental reflective functioning (PRF), ultimately leading to improved quality in carer-child relationships. Given the crucial role that foster carers’ emotional investment and quality of care play in promoting the well-being of children in their care, this study aims to assess the impact of COS-P on several key outcomes. This study will employ a mixed-methods randomized controlled trial (RCT) design, integrating quantitative and in-depth qualitative data. The following hypotheses will be tested:

#### Primary hypothesis (H1)

Foster carers who participate in the COS-P program will demonstrate a significant improvement in caregiving sensitivity at the 3-month follow-up assessment, as measured by the Caregiving Composite Questionnaire (CCQ). Specifically, we hypothesize that, compared to the waitlist control group, foster carers completing COS-P will, at the 3-month follow-up assessment, show.

(H1a) a greater increase in parental mentalizing,

(H1b) a greater increase in parenting self-efficacy, and.

(H1c) more positive perceptions of the child.

#### Secondary hypothesis (H2)

COS-P will enhance the quality of the relationship between foster carers and foster children, as evidenced by improvements in both dyadic interactions and overall relationship quality. Improvements in overall relationship quality will be assessed quantitatively using the Parenting Stress Index - IV - Short Form (PSI), while the Marschak Interaction Method (MIM) coded with the Dyadic Emotional Interaction Style (D-EIS) will be used to provide a complementary qualitative assessment of dyadic interactions. In addition to testing these hypotheses, the study will address the following objectives.

#### To explore potential moderators of intervention outcomes

This will include examining the influence of foster carer burnout, emotional support provided by Child Protection Services, and fidelity of program implementation.

#### To assess qualitatively the subjective experiences of participants

Focus groups and self-confrontation interviews will be conducted with foster carers and social workers who facilitated the COS-P program to gain a deeper understanding of their experiences with the intervention.

#### To assess potential iatrogenic effects

The study will carefully monitor for any unintended negative consequences of implementing COS-P within the french foster care system.

### Participants

#### Foster carers

The study will recruit foster carers from the 170 individuals employed by CPS and two accredited private foster care agencies in Haute-Loire, France (La Renouée and Mazel-Entraide Union). All foster carers will be offered the opportunity to participate in COS-P as part of their continuing education program. However, only those who meet the following criteria will be included in the study:

### Eligibility criteria

- Signed informed consent form.
- Commitment to attend all eight sessions of the COS-P program.
- Employed by CPS Haute-Loire or by one of the two participating private agencies.

#### Foster children

Children will participate in the dyadic interaction assessment.

### Eligibility criteria

- Placed with a foster carer participating in the study.
- Aged from 1 to 6 years.
- In permanent placement (5 or more days per week with the foster carer).
- Consent obtained from all individuals holding parental responsibility.

Recruitment will be completed by June 30, 2025.

#### Consent procedures

Two informational sessions will be held to present the COS-P training program and the research study. Foster carers will have the opportunity to provide consent online following these sessions. To obtain consent for the participation of children in the video-recorded assessments, placement services will contact those holding parental responsibility. Caseworkers will provide parents with a consent form, an informational brochure, and a link to an informational video.

### Study design and procedure

#### Randomized controlled trial design

This study will utilize a randomized controlled trial design with a mixed-methods approach. Participants will be randomly assigned to either the intervention group (receiving COS-P in addition to Treatment as Usual) or the waitlist control group (receiving Treatment as Usual only).

#### Treatment as usual (TAU)

Both groups will continue to receive Treatment as Usual (TAU), which consists of Psycho-Educational Support (PES) provided by CPS. PES typically involves home visits, educational and psychological support for foster carers and/or children in placement, and efforts to support and monitor contact between children and their birth families. The control group will be placed on a waiting list to receive the COS-P program as part of continuing education at a later date.

#### Circle of security parenting (COS-P) intervention

The intervention group will participate in the COS-P program in addition to TAU. Foster carers will be assigned to groups of 8 to 10 participants and will attend sessions in dedicated training facilities. The eight 2-hour sessions (detailed in Appendix 3) will be co-facilitated by two child protection professionals certified by Circle of Security International. If a participant misses a session, a 30-minute individual make-up session will be offered before the next group meeting.

#### Fidelity

To ensure fidelity to the COS-P model, all facilitators will be certified in the program after receiving four days of training. They will complete a fidelity checklist after each session, using the form provided in the intervention manual. Two group supervision sessions will be provided by a trainer accredited by Circle of Security International.

#### Randomization

A simple randomization procedure will be conducted at T0 by an independent researcher not involved in the study, using computer-generated random allocation.

#### Trial sites

Sessions will be held in child protection service facilities throughout the department to maximize accessibility and minimize travel time for participants (see Table 2).

**Table 2:** Trials sites

	Site 1 Community Health Centre, Brioude	Site 2 Child Protection Services, Saint-Georges d’Aurac	Site 3 Community Health Centre, Le Puy-en-Velay	Site 4 Community Health Centre, Yssingeaux
*Type of service*	Perinatal and public Health Foster care and family support for Chidren	Family and Group homes for Children Foster care and family support for children	Perinatal and public Health Foster care and family support for Chidren	Perinatal and public Health Foster care and family support for Chidren
*Profssionnal staff*	Nurses Midwives Social Workers Psychologists Pediatricians	Social workers Psychologists	Nurses Midwives Social Workers Psychologists Pediatricians	Nurses Midwives Social Workers Psychologists Pediatricians

### Data collection and security

Data acquisition and management will be conducted by trained researchers, including psychologists, research engineers, and master’s and doctoral level psychology students. All data will be anonymized or pseudonymized. Data will be collected from multiple sources: online questionnaires completed by foster carers, video recordings of interactions between foster carers and children, and anonymized transcripts derived from individual interviews and focus group discussions. For data security, all pseudonymized and anonymized data will be stored on secure servers specifically designed for the management of sensitive health information. All data management procedures will adhere to the standards established by the European General Data Protection Regulation (GDPR).

### Measures

#### Three-time measurement

Following an initial phase (T0), data collection will occur at three time points (see Table 3):

**T1 (Baseline)** Questionnaires, interviews, and interaction assessments will be administered in the week prior to the first COS-P session.

**T2 (3-month f/u)** Questionnaires, interviews, and interaction assessments will be administered three months after the final COS-P session.

**T3 (Qualitative Data Collection at 4-month f/u)** Focus groups will be conducted four months after the completion of the intervention (see Table 4 for an overview of assessments).

**Table 3:** SAFE FAMILIES study data collection schedule

	(T0) Enrollment and allocation	(T1) Baseline	COS-*P* group (intervention arm only)	(T2) 3-month f/u	(T3) 4-month f/u
Foster Parent Consent	X				
Biological Parent Consent (video recording)	X				
Sociodemographic and Professional Questionnaire	X				
*Quantitative assessments*					
CCQ		X		X	
PSI		X		X	
PBA		X		X	
SAQ		X		X	
MSPE		X		X	
MIM-DEIS		X		X	
PDI-R		X		X	
COS-P Fidelity Log			X		
*Qualitative assessments*					
Self-Confrontation Interviews		X		X	
Focus-groups					X

**Table 4:** Overview of qualitative and quantitative assessments

	Control Group (Gc) + Intervention Group (Gi) *N* = 70	Subgroups of Gi
	Quantitative assessments	Qualitative assessments
**Primary Outcome** Assess foster parent caregiving sensitivity and reflective functioning	CCQ	PDI-R (*N* = 20) Self-confrontation Interviews (*N* = 6)
**Secondary Outcome** Assess the relationship between the foster parent and the child		
	PSI	SMRC (*N* = 20), MIM-DEIS (*N* = 20) Focus Groups
**Exploratory Outcomes**		
Investigate the role of parental burnout and emotional support	PBA SAQ	Focus Groups, Self-Confrontation Interviews (*N* = 6)
Evaluate participants’ subjective experiences		

#### Primary outcome

**Caregiving composite questionnaire (CCQ).** The CCQ is a 43-item self-report measure specifically designed to assess parent capacities targeted by the COS-P program [28], namely: parental mentalizing, parenting self-efficacy, and parent perceptions of the child. It combines six subscales and one single item drawn from previously validated measures, retaining the original response formats and scoring procedures for each subscale. The subscales are: (a) Parental Mentalizing (18 items; e.g., *I think about what my child may be thinking or feeling*; α = 0.81); (b) Parenting Self-Efficacy - Empathy (6 items; e.g., *I am able to put myself in my child’s shoes*; α = 0.87); (c) Parenting Self-Efficacy - Affection (9 items; e.g., *I am able to show affection to my child*; α = 0.75); (d) Caregiving Helplessness (7 items; e.g., *When I am with my child*,* I often feel out of control*; α = 0.86) and (e) Parent Perceptions of the Child - Hostility (5 items; e.g., *When this child cries*,* he/she gets on my nerves*; α = 0.89. The CCQ has demonstrated good reliability and validity in a pilot sample of parents [29] and is being used in the current study to assess the impact of COS-P on these key parenting capacities. This questionnaire has also undergone validation with a sample of *N* = 1181 French foster carers as part of a larger research project on foster care conducted by our research team. Results are currently being prepared for publication.

**Parent Development Interview – Revised (PDI-R).** This semi-structured interview consists of 31 questions, administered in approximately one hour, covering four domains: (a) perception of the child (e.g., “What are some of your child’s strengths?”), (b) perception of the relationship (e.g., “What are some of the things you enjoy doing together?”), (c) parental affective experience (e.g., “How does it feel to be separated from your child?”), and (d) separation experiences (e.g., “Tell me about experiences of separation from your own parents when you were a child”). These domains assess reflective functioning (RF) in relation to the self (as a foster carer in this context) and the child. Parental reflective functioning (PRF) will be rated using the system developed by Slade et al. [30–32], on a scale from 1 (negative reflective functioning: rejection of mental states) to 9 (exceptional reflective functioning). The version used in this study is the French translation by Ensink et al. [33], following the translation/back-translation method described by Behling and Law [34]. The PDI-R is a well-validated measure of PRF and will be administered to a subgroup of foster carers.

#### Secondary outcome

**Parenting stress index – IV – short form (PSI).** Developed by Abidin [35, 36], this 36-item questionnaire includes three subscales: Parental Distress (PD; e.g., “I feel trapped by my responsibilities as a parent”), Parent-Child Dysfunctional Interaction (P-CDI; e.g., “I often have the feeling that my child is misbehaving on purpose”), and Difficult Child (DC; e.g., “My child rarely does things that make me happy”).Touchèque et al. [37] reported alpha coefficients of 0.79 for each of the three subscales (PD, P-CDI, and DC) in a French sample. The 12 items of the P-CDI subscale will be used in this study to assess foster carers’ perceptions of their relationship with the child in their care, using a 5-point Likert scale (1 = Strongly Disagree, 2 = Disagree, 3 = Not Sure, 4 = Agree, 5 = Strongly Agree).

**Marschak interaction method (MIM).** This structured assessment tool is based on attachment and mentalization theories and involves a series of age-appropriate tasks completed by the foster carer and child together [38, 39]. The MIM assesses four dimensions of interaction: Structure, Engagement, Nurture, and Challenge. Interaction recordings will be coded using the Dyadic Emotional Interaction Style (D-EIS), developed in Finland [40] and used internationally. The D-EIS consists of nine items, reflecting the four MIM dimensions, rated on a continuum from 0 (indicating significant difficulties requiring clinical intervention) to 5 (reflecting a very positive relationship not requiring intervention). This coding system yields a total score ranging from 0 to 40, integrating observations of both the child and the caregiver [41]. The MIM manual has been translated into French by certified coders from our research team, with permission from the original developer. In this study, a subgroup of dyads will be assessed using the MIM and D-EIS coding system, with all coding conducted using a double-blind procedure.

**Subjective Measure of Relational Closeness (SMRC).** This measure, developed specifically for this study, includes two items that will be administered to foster carers after completing the MIM: (1) “How emotionally close do you feel to the child currently in your care?” and (2) “How important do you think you are to the child currently in your care?” Each item will be rated on a 5-point Likert scale, ranging from “not at all” to “extremely.”

#### Exploratory outcomes measures

**Parental burnout assessment (PBA).** This 23-item questionnaire assesses parental burnout [42] across four dimensions using a 7-point Likert scale (1 = *Never*, 2 = *Almost never*, 3 = *Sometimes*, 4 = *Regularly*, 5 = *Often*, 6 = *Very often*, 7 = *Always*): (a) exhaustion (e.g., “I feel completely run down by my parenting role”), (b) contrast with previous experience (e.g., “Being a parent is much more difficult than I could have ever imagined”), (c) sense of being overwhelmed in the parenting role (e.g., “I can’t stand my role as a parent anymore”), and (d) emotional distancing from one’s children (e.g., “I have no fun being with my children anymore”). The PBA demonstrates strong internal consistency: α = 0.96 for the total score, 0.93 for exhaustion, 0.90 for contrast and saturation, and 0.79 for emotional distancing [43]. This validation study included a sample of both French-speaking and English-speaking parents (*N* = 901), thus validating the PBA for use with French-speaking populations.

**Service Attachment Questionnaire (SAQ).** This 25-item questionnaire was originally designed to assess attachment security between patients and mental health services [44]. It will be adapted for this study to assess the quality of the relationship between foster carers and their CPS, focusing on five key themes: focusing on five key themes derived from the original six SAQ subscales: (a) feeling listened to (e.g., “I have somebody who listens attentively to me.”), (b) experiencing continuity in the helping relationship (e.g., “I have regular time with the same person that knows me and my problems.”), (c) receiving adequate time and attention (e.g., “I feel under pressure to get better and be discharged.” [reverse-scored]), (d) feeling accepted (e.g., “I feel that people at CPS understand my needs and problems.”), and (e) finding comfort in times of difficulty (e.g., “I have a feeling of being looked after.”). The original SAQ demonstrated good internal consistency (α = 0.93) and test-retest reliability (mean *r* =.74 across subscales) in a sample of 154 service users. This questionnaire has also undergone validation with a sample of *N* = 1181 French foster carers as part of a larger research project on foster care conducted by our research team (validation article in preparation).

**Fidelity of implementation** (**COS-P Fidelity Log).** This log consists of 12 Likert-scale items per session (96 items in total) and reflective questions, allowing facilitators to self-assess the fidelity and quality of program implementation with their group. For the purposes of this study, only the 96 Likert-scale items will be used for data analysis.

### Qualitative measures

**Focus groups.** Two types of focus groups [45, 46] will be conducted:

*(a) With COS-P facilitators.* These focus groups will explore the subjective experiences of the social workers who facilitated the COS-P program. Drawing on their professional expertise and observations, facilitators will be asked to reflect on the appropriateness of the program for foster carers and the feasibility of implementing it within the French child protection system.

*(b) With foster carers.* Eight foster carers who participated in the COS-P intervention will be invited to participate in focus groups to discuss their experiences. These discussions will explore the perceived benefits and challenges of the program, and foster carers will be asked to share their perspectives on whether COS-P should be incorporated into pre-service or continuing education for foster carers. Participant recruitment will aim for diversity in terms of the age of children in their care, years of experience as a foster carer, and the number of children in the home. These focus groups will also provide valuable qualitative data relevant to the study’s complementary objectives, particularly regarding the foster carers’ experiences of emotional support and any potential iatrogenic effects of the intervention.

**Self-Confrontation Interviews.** Six foster carers will be invited to participate in self-confrontation interviews [47], in which they will view a video recording of themselves and their foster child participating in the MIM. This method will allow for in-depth exploration of the foster carers’ subjective experiences of the assessment and the impact of COS-P on their understanding of their foster child, their relationship with the child, and their approach to caregiving.

### Risks and iatrogenic effects

The COS-P intervention has been widely implemented in Europe, the United States, Australia, and other countries, and has been translated into eight languages. To date, no associated risks or negative effects have been reported [48]. A research monitoring committee composed of researchers and representatives from the Départment of Haute-Loire and accredited foster care agencies, will oversee the study to ensure ethical conduct and the well-being of all participants. Importantly, participation in the study will not affect foster carers’ or children’s access to usual psycho-educational support services.

### Statistical analyses

***Sample size and analysis.*** Sample size calculation was based on an effect size of *d* = 0.38, drawn from a meta-analysis examining the impact of attachment-based interventions on parental sensitivity [49]. An a priori power analysis using G*Power software [50] indicated that 58 participants are needed to achieve 80% power with a significance level of *p* <.05. To account for potential attrition (20%), we aim to recruit 35 participants for each group (intervention and waitlist control receiving TAU).

The data will be analyzed using both quantitative and qualitative methods. Statistical analyses will be conducted using R software. All tests will be two-tailed with a significance level set at *p* <.05.

***Quantitative analyses.*** Descriptive statistics will be used to characterize the sample at baseline (T1) and at each assessment point (T2, T3). This will include frequencies and percentages for categorical variables (e.g., gender) and means and standard deviations for continuous variables (e.g., age, duration of placement). To test the primary hypothesis (H1), a two-way mixed ANOVA will be conducted with Group (Intervention vs. Control) as the between-subjects factor and Time (T1, T2) as the within-subjects factor. The dependent variable will be the CCQ score. To test the secondary hypothesis (H2), a two-way mixed ANOVA will be conducted with Group (Intervention vs. Control) as the between-subjects factor and Time (T1, T2) as the within-subjects factor. The dependent variable will be the PSI score. To explore the potential moderating effects of parental burnout (PBA), perceived emotional support from placement services (SAQ) and fidelity of program implementation, hierarchical multiple linear regressions will be performed. The CCQ and PSI scores at T2 will be the dependent variables, with group allocation (Intervention vs. Control) as the primary predictor. The potential moderators (PBA, SAQ, and fidelity of implementation) will be entered in the model after the main effect of group allocation.

Complementary Analyses for a Subgroup of Foster Carers: A subgroup of 20 foster carers will participate in additional assessments using the Parent Development Interview - Revised (PDI-R) and the Marschak Interaction Method (MIM) coded with the Dyadic Emotional Interaction Style (D-EIS). These assessments will be conducted at T1 (baseline) and T2 (3-month follow-up).

Paired-samples t-tests will be used to compare the PDI-R and MIM-DEIS scores at T1 and T2 for the subgroup of foster carers. These analyses will explore the impact of the COS-P program on parental reflective functioning (as measured by the PDI-R) and on the quality of emotional dyadic interactions (as measured by the MIM-DEIS subscales) within this subgroup of foster carers.

***Qualitative analyses.*** Data from the self-confrontation interviews and focus groups will be analyzed using the Braun and Clarke thematic analysis approach [51]. This will involve a systematic process of identifying, coding, and categorizing patterns within the data. The aim is to develop a comprehensive understanding of participants’ experiences with the COS-P program and its perceived impact. Thematic analyses will be conducted using NVivo software.

***Data triangulation.*** Findings from the quantitative and qualitative analyses will be compared and contrasted to provide a richer and more nuanced understanding of the effects of COS-P in the context of foster care. This triangulation approach will enhance the validity and trustworthiness of the study findings.

**Fig. 3:**
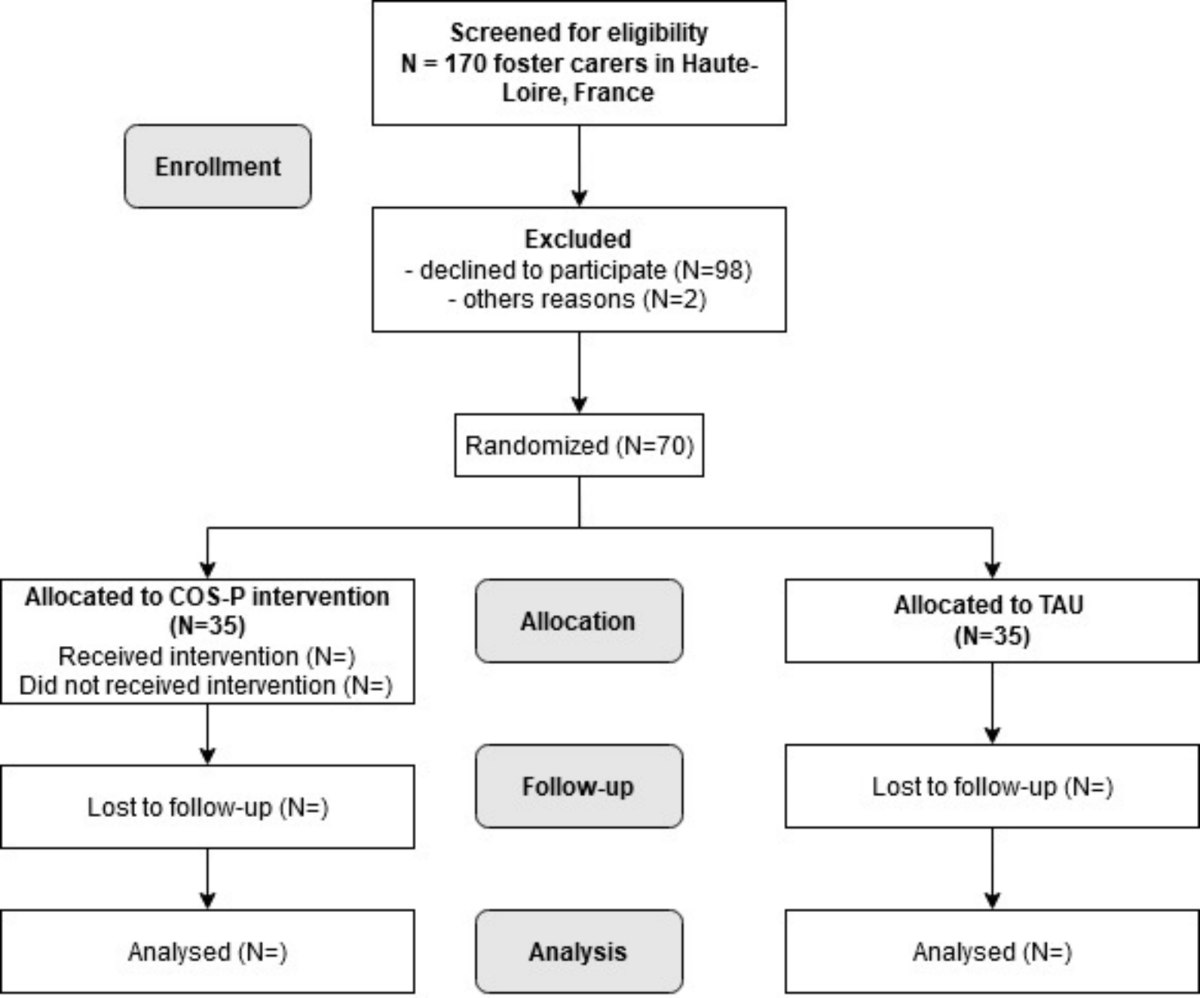
CONSORT diagram describing flow of participants through study

## Discussion

This study protocol outlines a mixed-methods randomized controlled trial designed to evaluate the effectiveness of the Circle of Security Parenting program^®^ (COS-P) for foster carers in France. The study aims to demonstrate the program’s impact on several key outcomes. Primarily, it seeks to assess whether COS-P can improve foster carers’ caregiving sensitivity, particularly their parental reflective functioning. Secondly, it will examine if COS-P enhances the quality of the relationship between foster carers and foster children. Additionally, the study will explore potential moderating factors, such as foster carer burnout and perceived support from child protective services (CPS), and delve into participants’ subjective experiences with the program.

This study addresses a critical gap in the literature by conducting the first evaluation of COS-P specifically for foster carers in France. While previous research has established the efficacy of COS-P in improving parenting outcomes in other populations, including mothers and residential care workers, this study will provide valuable insights into the program’s applicability and effectiveness within the unique context of the French child protection system.

The study boasts several strengths, including its randomized controlled trial design. Furthermore, the use of validated and reliable measures strengthens the study’s methodological rigor. By incorporating both quantitative and in-depth qualitative data, the study aims to provide a comprehensive and nuanced understanding of the program’s impact.

However, the study also acknowledges certain limitations. Firstly, its geographic scope is limited to one region in France, which may constrain the generalizability of the findings. Secondly, the relatively short follow-up period may not fully capture the long-term impact of the intervention. Finally, a potential for selection bias exists. Foster carers who choose to participate in the study might already be more engaged in their relationships with foster children and more emotionally supportive compared to those who decline participation. This pre-existing difference could lead to an underestimation of the program’s effects on caregiving sensitivity.

Despite these limitations, findings from this study hold significant implications for policy and practice within the French child protection system. Should COS-P prove effective, it could be integrated into pre-service or continuing education programs for foster carers. Such integration has the potential to enhance the quality of care provided to children in out-of-home placements, ultimately contributing to improved outcomes for this vulnerable population. Furthermore, the study’s findings could inform the development of tailored interventions that address the specific needs of foster carers, including interventions that target burnout and bolster support networks. Looking ahead, future research should prioritize exploring the long-term impact of COS-P and delve into its effectiveness across different cultural contexts.

## Data Availability

No datasets were generated or analysed during the current study.

## Abbreviations

CCQ: Caregiving Composite Questionaire
CEEI-IRB: Comité d’éthique et d’évaluation de l’INSERM- Institutional Review Board
CONSORT: Consolidating Standards of Reporting Trials
COS-I: Circle of Security-Intensive
COS-P: Circle of security-parenting
CPS: Child Protection Services
DEIS: Dyadic Emotional Interactive Style
GDPR: General Data Protection Regulation
INSERM: Institut National de la Santé et de la Recherche Médicale
MIM: Marschak Interaction Method
ONPE: Observatoire Nationale de la Protection de l’Enfance
PBA: Parental Burnout Assessment
PDI-R: Parent Development Interview-Revised
PES: Psycho-Educational Support
PRF: Parental Reflective Functionning
PSI: Parenting Stress Index
RCT: Randomized Controlled Trial
SAQ: Service Attachment Questionaire
SMRC: Subjective Measure of Relational Closeness
TAU: Treatment as Usual
